# Preparing for the primary care clinic: an ambulatory boot camp for internal medicine interns

**DOI:** 10.3402/meo.v20.29702

**Published:** 2015-11-24

**Authors:** Lindsay M. Esch, Amber-Nicole Bird, Julie L. Oyler, Wei Wei Lee, Sachin D. Shah, Amber T. Pincavage

**Affiliations:** 1 Internal Medicine Residency, University of Chicago, Chicago, IL, USA; 2 Department of Medicine, University of Pennsylvania, Philadelphia, PA, USA; 3 Department of Medicine, University of Chicago, Chicago, IL, USA; 4 Department of Medicine & Pediatrics, Medicine-Pediatrics Residency, University of Chicago, Chicago, IL, USA

**Keywords:** boot camp, resident continuity clinic, ambulatory training, internal medicine residency training, resident primary care clinic

## Abstract

**Introduction:**

Internal medicine (IM) interns start continuity clinic with variable ambulatory training. Multiple other specialties have utilized a boot camp style curriculum to improve surgical and procedural skills, but boot camps have not been used to improve interns’ ambulatory knowledge and confidence. The authors implemented and assessed the impact of an intern ambulatory boot camp pilot on primary care knowledge, confidence, and curricular satisfaction.

**Methods:**

During July 2014, IM interns attended ambulatory boot camp. It included clinically focused case-based didactic sessions on common ambulatory topics as well as orientation to the clinic and electronic medical records. Interns anonymously completed a 15-question pre-test on topics covered in the boot camp as well as an identical post-test after the boot camp. The interns were surveyed regarding their confidence and satisfaction.

**Results:**

Thirty-eight interns participated in the boot camp. Prior to the boot camp, few interns reported confidence managing common outpatient conditions. The average pre-test knowledge score was 46.3%. The average post-test knowledge score significantly improved to 76.1% (*p<*0.001). All interns reported that the boot camp was good preparation for clinics and 97% felt that the boot camp boosted their confidence.

**Conclusions:**

The ambulatory boot camp pilot improved primary care knowledge, and interns thought it was good preparation for clinic. The ambulatory boot camp was well received and may be an effective way to improve the preparation of interns for primary care clinic. Further assessment of clinical performance and expansion to other programs and specialties should be considered.

Medical education in internal medicine (IM) residency traditionally focuses on the inpatient setting, with an inadequate emphasis on ambulatory education (1). It has also been noted that ambulatory education is considered to be less valuable to residents than inpatient-based education (2). However, most IM residents will eventually spend a large portion of their time after residency training taking care of patients in the ambulatory setting, and in recent years there has been a movement to reform and improve ambulatory training in IM (1).

Although there has been documentation of the ‘July effect’, where inpatient care is known to suffer during the early part of July as new interns begin their training, this has not been examined in the ambulatory setting (3). However, there is variable exposure to ambulatory and primary care medicine in medical school and although inpatient subinternships are required during the fourth year of medical school, ambulatory training during this final year can be very limited. Thus, it is likely many interns begin IM training with more remote primary care training than inpatient training and may be poorly prepared to begin resident continuity clinic. Even senior IM residents report low preparedness to treat common outpatient conditions compared to inpatient conditions, and their preparedness is associated with their exposure to outpatient settings (4). Thus, further study into the ambulatory preparedness of new IM interns and how best to prepare IM interns for continuity clinic is needed.

Boot camps have been utilized in various arenas to help prepare trainees for new roles and duties. They have been used with fourth-year medical students and residents to improve confidence and performance in procedural and communication skills (5–10). Moreover, boot camps have also been used in a variety of specialties including emergency medicine, IM, neurosurgery, orthopedics, obstetrics and gynecology, and several surgical subspecialties. One IM boot camp for interns that focused on improving management of critically ill patients, cardiac auscultation skills, code status communication, and procedural skills successfully improved intern confidence in these skills and performance on competence testing (10).

Although boot camps have been widely used to prepare trainees for the inpatient setting and procedures, they have not been implemented in ambulatory education, or specifically to prepare IM interns for their resident continuity clinic. Our aim was to implement and assess the impact of an intern ambulatory boot camp pilot on primary care knowledge, confidence, and curricular satisfaction, and to ultimately determine if an intern ambulatory boot camp may be an effective way to prepare IM interns for primary care clinic.

## Methods

The intern ambulatory boot camp was implemented in July 2014. Thirty-eight new postgraduate year-1 IM interns, both categorical and preliminary, at the University of Chicago Medical Center were scheduled to participate in the boot camp prior to the beginning of ambulatory training.

The University of Chicago IM program utilizes a ‘4 + 2’ block scheduling model, in which all interns and residents have 4 weeks of inpatient responsibilities followed by a 2-week block dedicated to outpatient responsibilities, which includes IM ambulatory continuity clinic, urgent care clinic, emergency medicine, subspecialty clinics, consult experiences, and didactics. The boot camp was conducted during the interns’ first ‘ + 2’ ambulatory block prior to starting continuity clinic or other ambulatory experiences.

This study was reviewed and granted exemption status by the Institutional Review Board at the University of Chicago prior to the initiation of the study.

The boot camp included a 2-day timeline (Table 1). Because we thought interns needed most help with their ambulatory medical knowledge, we focused mainly on improving their medical knowledge with more traditional classroom methods. During the first day morning session, interns participated in five clinically focused, case-based didactic sessions on five common topics seen in IM primary care clinic: diabetes, hypertension, hyperlipidemia, musculoskeletal complaints focusing on the shoulder and knee, and health maintenance screening. The afternoon of the first day consisted of a tour of the clinic, a clinic orientation, a session on ambulatory electronic medical record (EMR) training basics, and an interprofessional learning session. The interprofessional learning session consisted of a brief introduction to the clinic staff with a face sheet of their pictures and titles. Interns spent 20 min, one-on-one with a clinic staff member (medical assistants, registered nurses, licensed practice nurses, pharmacists, check-in, and check-out staff) getting to know them and watching their interactions with patients to understand their role. Interns then summarized what they learned for the rest of the group. The second day morning session consisted of workshops on communication skills to optimize patient-centered EMR use and an additional ambulatory EMR training lecture. The second day afternoon session consisted of a half day of clinic (four patient encounters) or other ambulatory experience, depending on the interns’ assigned rotation.

**Table 1:** Intern ambulatory boot camp schedule

	Day 1	Day 2
Morning	-Pre-test/survey	-Patient-centered EMR use lecture
	-Lectures/case discussions	-Clinic EMR tips lecture
	-Diabetes	-Survey
	-Hypertension	
	-Hyperlipidemia	
	-Musculoskeletal	
	-Health maintenance	
	-Post-test/survey	
Afternoon	-Clinic tour	-Full half day of ambulatory clinic (4 patients)
	-Ambulatory EMR training	or other outpatient experience
	-Interprofessional learning	

On the first day morning prior to starting boot camp, all interns completed an anonymous 15-question multiple choice knowledge exam testing the topics covered: diabetes, hypertension, hyperlipidemia, musculoskeletal complaints focusing on the shoulder and knee, and health maintenance. The test contained approximately three case-based questions per topic. An identical knowledge exam was given at the conclusion of the first day to assess for improvement in knowledge. The interns also answered anonymous survey questions both immediately before and after the boot camp to assess prior ambulatory experience, confidence evaluating and managing common conditions, and satisfaction with the boot camp (Supplementary files). Interns were surveyed regarding the patient-centered EMR use workshop on the second day. Interns were surveyed again 2 months after the boot camp to obtain their perspectives after more extensive primary care exposure (Supplementary file). Most of the survey items were formatted as a statement to which the participant responded using a five-point Likert scale in which responses ranged from strongly disagree to strongly agree.

Using Stata version 12.0 (Stata Corp., College Station, TX), we used descriptive statistics to summarize baseline participant characteristics, exam data, and survey data. We dichotomized resident survey data for analysis (we defined ‘agree’ as a Likert response of either agree or strongly agree). We used the Student's *t*-test for analysis of the pre-test and post-test scores as well as other statistical analyses requiring comparison among two groups.

## Results

All 38 (100%) of IM interns participated in boot camp and completed the immediate surveys and knowledge tests. The average age of participants was 26.9 years, and 15 (39.5%) were male. The interns reported an average of 2.9 months of primary care rotations during medical school (ranging from less than 1 month to 12 months). On average, interns reported 15 months had elapsed since their last ambulatory care experience (ranging from 4 months to 26 months).

The average pre-test score was 6.95/15 (46.3%). This improved after the boot camp to an average post-test score of 11.42/15 (76.1%; *p<*0.001). There was no difference in the pre-test score average if the intern had less than 3 months of ambulatory rotations during medical school or if they had 3 or more months of ambulatory rotations during medical school (*p*=0.58). In addition, there was no difference in pre-test score average if the last primary care rotation was more than 6 months ago or less than 6 months ago (*p*=0.56), or even if the last primary care rotation was more than 12 months ago or less than 12 months ago (*p*=0.46).

Prior to boot camp, interns did not feel confident evaluating and managing the five common ambulatory topics included in the boot camp (Table 2). Interns also consistently noted that they had not received enough ambulatory training in medical school. After completing boot camp, interns noted it was good preparation for ambulatory clinic, and also thought it should be a required component of internship (Table 3). Interns reported increased confidence managing common conditions and felt that because of the boot camp, they would be able to provide better care to their patients.

**Table 2:** Pre boot camp survey items

	Mean (SD)	Agreement, *n* (% of 38)
I feel confident evaluating and managing diabetes in an outpatient clinic setting	3.1 (0.8)	12 (31.6%)
I feel confident evaluating and managing hypertension in an outpatient clinic setting	3.5 (0.8)	21 (55.3%)
I feel confident evaluating and managing hyperlipidemia in an outpatient clinic setting	3.3 (0.8)	19 (50.0%)
I feel confident evaluating and managing recommended routine health maintenance screening in an outpatient clinic setting	3.3 (0.8)	16 (42.1%)
I feel confident evaluating and managing common musculoskeletal complaints in an outpatient clinic setting	2.2 (0.9)	4 (10.5%)
I feel that I received sufficient training in primary care during medical school to manage patients independently in a primary care clinic	2.7 (0.8)	6 (15.8%)
I feel that an intern ambulatory boot camp will be good preparation for ambulatory clinic in internal medicine internship	4.5 (0.6)	35 (92.1%)
I feel that I have received sufficient training during medical school in communicating with nurses and clinic staff	3.8 (0.8)	25 (65.8%)

Residents were surveyed regarding their agreement based on a five-point Likert scale in which responses ranged from strongly disagree (1) to strongly agree (5). Resident survey data were dichotomized for analysis (we defined ‘agree’ as a Likert response of either agree or strongly agree).

**Table 3:** Immediate post boot camp survey items

	Mean (SD)	Agreement, *n* (% of 38)
Intern ambulatory boot camp was good preparation for ambulatory clinic in internal medicine internship	4.7 (0.5)	38 (100.0%)
Educational lectures were engaging	4.5 (0.7)	35 (92.1%)
I learned useful information from the training sessions	4.8 (0.4)	38 (100.0%)
Ambulatory intern boot camp should be a required component of internship	4.8 (0.4)	38 (100.0%)
Lecture sessions on selected ambulatory topics boosted my confidence in managing common conditions encountered in the ambulatory clinic	4.7 (0.5)	37 (97.3%)
The educational sessions will allow me to provide better care for my clinic patients	4.7 (0.5)	38 (100.0%)
Intern ambulatory boot camp helped me learn to communicate with nurses and clinic staff	3.2 (1.3)	13 (34.2%)

Residents were surveyed regarding their agreement based on a five-point Likert scale in which responses ranged from strongly disagree (1) to strongly agree (5). Resident survey data were dichotomized for analysis (we defined ‘agree’ as a Likert response of either agree or strongly agree).

Thirty-three interns (87%, 33/38) completed the post-survey after the patient-centered EMR use workshop. Most interns agreed that the workshop should be required for all residents (88%, 29/33). Overall, the majority of interns (94%, 31/33) agreed that the workshop will help them better integrate the EMR into their workflow and communication with patients.

Twenty-eight interns (74%, 28/38) completed the 2-month post-survey. The majority (89%, 25/28) still agreed that boot camp had been good preparation for ambulatory clinic. Also, most interns (86%, 24/28) reported that they had already applied information they learned in the boot camp to their clinic patients. In addition, the majority of interns (82%, 23/28) agreed that the session on how to effectively use the EMR was good preparation for ambulatory clinic.

Representative comments left by the interns included: ‘the lectures were a great intro into outpatient medicine and I think they should be included in every orientation’ and ‘I found these lectures to be very informative. It was a lot of information but a good review and I learned quite a bit’. Specific suggestions for improvement included spacing the information over a few more days and having more information about navigating the clinic.

## Discussion

The IM intern ambulatory boot camp was easy to implement without the need for additional funding. It only required willing and capable instructors to prepare the lectures and present the material and 2 days of protected time for completion. It was well received by interns and perceived to be helpful. Specifically, the boot camp increased the knowledge of ambulatory topics, improved intern confidence in managing common ambulatory topics, and helped integration of the EMR into their workflow. Two months after starting continuity clinic, interns still felt that the ambulatory boot camp was good preparation.

Baseline primary care knowledge among new IM interns was poor in general and confidence in ambulatory medicine was low, demonstrating a need for the ambulatory boot camp. Interestingly, we found that pre-test scores did not differ based on the number of months of prior primary care rotations during medical school or the amount of time that had elapsed since the interns’ most recent primary care rotation. This may have been partially due to the small sample size or that the duration from last primary care exposure was at least 4 months for all of the interns.

The interns did not think the interprofessional learning helped them learn to communicate with nurses and clinic staff. This may be partially due to the limited duration of the interprofessional learning experience or that interns reported greater training in this communication in medical school at baseline. It is likely that more extensive interprofessional education and training will be required to improve these high-level skills.

There are limitations to this study. This curriculum included a small sample size of interns, and the curriculum has only been implemented for 1 year. Although objective data were collected to assess improvement in knowledge, no data were collected regarding improvement in ambulatory clinical skills or clinical performance. We also did not assess for knowledge retention over weeks to months. It is possible that our positive findings were partially attributable to the timing of the testing immediately after the session. The boot camp design, presenting a large amount of information over a small amount of time, can limit knowledge retention. In addition, because ‘boot camps’ are time limited by nature, there is only a limited amount of information that can be presented.

Future directions could include retesting interns with another post-test weeks or months after the boot camp to assess knowledge retention. Assessment of the boot camp impact by direct observation of clinical skills in clinic or with standardized patients should be pursued. In addition, the boot camp could be expanded to include common clinic procedures, such as joint injections or pap smears. Also, including a simulated clinical immersion experience with standardized patients should be considered. Further expansion of interprofessional learning would be beneficial as well. Implementation of boot camps for ambulatory education in other specialties and other IM residency programs should also be pursued to see if improvements in ambulatory knowledge and confidence are obtained. In addition, other models of training to help prepare IM interns for continuity clinic should be tried and studied.

## Conclusions

The IM intern ambulatory boot camp improved intern knowledge of common ambulatory topics, as well as intern confidence prior to starting continuity clinic. Thus, an ambulatory boot camp is a feasible and effective way to prepare IM interns for the resident primary care clinic. Application of an ambulatory boot camp in other residency programs should be considered.

## Supplementary Material

Preparing for the primary care clinic: an ambulatory boot camp for internal medicine interns
