# Supplementary material for: Preparing for the primary care clinic: an ambulatory boot camp for internal medicine interns
Source: Med Educ Online. 2015 Nov 24;20:10.3402/meo.v20.29702. doi: 10.3402/meo.v20.29702 (PMC4660998; doi:10.3402/meo.v20.29702)
Supplement: Preparing for the primary care clinic: an ambulatory boot camp for internal medicine interns [file MEO-20-29702-s001.pdf]

Internal Medicine Ambulatory Curriculum Pre-Test/Survey

Please answer the following questions to the best of your ability. Each question has one best answer. Please do not write your name on this test. We will be using the anonymous results for research purposes. Participating is completely voluntary. Thanks for your participation!

1. Ms. Smith is an 88 year old woman that you are seeing during a follow up clinic appointment. She has type 2 diabetes mellitus and you have been working with her to get her diabetes optimally controlled. Currently she takes metformin 1000 mg bid. She has been stable on this regimen for 2 years. She also has a history of myocardial infarction and congestive heart failure secondary to coronary artery disease. Her left ventricular ejection fraction is 15%. She is wheel-chair bound and lives at a nursing home. Her hemoglobin A1C checked on today's blood work is 7.8. Her random blood glucose in your office today is 188. Which of the following is the most appropriate statement regarding her hemoglobin A1C goal?
    a. Her goal hemoglobin A1C is <6.5. Another oral medication, such as a sulfonylurea, should be added and the hemoglobin A1C should be rechecked in 3 months.
    b. Her goal hemoglobin A1C is <7.0. Long acting insulin at bedtime should be added and the hemoglobin A1C should be rechecked in 1 month.
    c. Her goal hemoglobin A1C is <7.0. Another oral medication, such as a sulfonylurea, should be added and the hemoglobin A1C should be rechecked in 3 months.
    d. Her goal hemoglobin A1C is <8.0 in the setting of her multiple comorbidities and limited life expectancy. Current management is appropriate. A repeat hemoglobin A1C should be rechecked in 6 months.

2. Which of these comorbid conditions commonly found in diabetes is matched up correctly with the current comorbidity screening recommendations in diabetic (type II) patients?
    a. Nephropathy: Spot urine albumin-to-creatinine ratio every 2 years
    b. Neuropathy: No screening recommended
    c. Retinopathy: Full eye exam every 2 years
    d. None of the above

3. Mr. Kale is a 53 year old man with type 2 diabetes mellitus. He has been on metformin 1000 mg bid and glipizide extended release 20 mg daily for the past 1 year. He tells you at his clinic visit that he is checking his blood glucose every morning before eating. For the past 3 months, his morning glucose values average around 200 and are never below 150. He reports compliance with his medications, as well as a diet and exercise routine. His weight in clinic today is 100 kg. His hemoglobin A1C today is 9.7. Which of the following is the most appropriate next step in management to get his A1C at goal?
    a. Add Januvia (sitagliptin) 100 mg once daily in addition to the metformin and glipizide
    b. Start glargine 20 units at bedtime, continue metformin but discontinue the glipizide
    c. Start glargine 20 units at bedtime, continue the metformin and the glipizide
    d. Start Humalin 70/30 (insulin NPH/insulin regular) 20 units twice daily, continue the metformin and the glipizide

4. A 55 year old man comes for an office visit after he was recently diagnosed with type 2 diabetes. He has no history of heart or renal disease. On physical exam, his blood pressure is 138/86 mm Hg, heart rate is 80 bpm, and respiratory rate is 16 breaths/min. His BMI is 29. The remainder of his physical exam is normal. Laboratory studies including electrolytes, creatinine, and BUN are normal. His hemoglobin A1C is 10.0. His urine albumin/creatinine ratio is 20 mg/g. What is the maximal allowable target blood pressure for this patient?
    a. Less than 115/75 mm Hg
    b. Less than 125/75 mm Hg
    c. Less than 130/80 mm Hg
    d. Less than 140/90 mm Hg

5. Ms. Jones comes into your clinic as a new patient. She is a healthy 51 year old woman with a past medical history of osteoarthritis and obesity who was referred to you for elevated blood pressure. She was seen by Gynecology 2 weeks ago for her routine pap smear and was found to have a blood pressure of 162/100, although she felt fine. She was also seen by Rheumatology 3 days ago for evaluation of her osteoarthritis and was found to have an elevated blood pressure at that visit as well, 168/96. Her blood pressure today is 160/92. She reports that she has been checking her blood pressure at home as well and she gets similar readings. You do a very thorough history and physical exam and decide that she likely has primary essential hypertension. Which of the following studies are indicated in the routine evaluation of a patient with newly diagnosed hypertension?
    a. EKG, blood chemistries, lipid panel, urinalysis
    b. Blood chemistries, lipid panel, thyroid studies
    c. Blood chemistries, lipid panel, transthoracic echocardiogram
    d. EKG, blood chemistries, thyroid studies, urinalysis

6. Which type of anti-hypertensive is currently recommended as the first line anti-hypertensive in a nonblack otherwise healthy adult?
    a. Thiazide
    b. Ace inhibitor/Angiotensin receptor blocker
    c. Calcium channel blocker
    d. All of the above

7. A 60 year old man with type 2 DM is seen in PCG to establish medical care. Is other medical problems include HTN and he is a current smoker, smoking 1 pack per day for the past 40 years. His daily medications are metformin, aspirin, and hydrochlorothiazide. On physical examination, his blood pressure is 125/75 mm Hg and his pulse is 80 bpm. His BMI is 27. The remaining physical examination findings are normal. Labs are shown below:

Total cholesterol	160 mg/dL
HDL	60 mg/dL
LDL	64 mg/dL
Triglycerides	180 mg/dL
Hemoglobin A1C	6.5%

Which of the following drugs should be initiated, if any?
    a. No therapy indicated, patient’s LDL is at goal
    b. Simvastain 20 mg daily
    c. Atorvastatin 80 mg daily
    d. Niacin 250 mg daily

8. A 40 year old African American man is evaluated at a follow up visit in your clinic. He had a fasting lipid panel performed 3 weeks ago at your last visit. He does not smoke, has no history of heart attack or stroke, no diabetes. He is a healthy, active man and exercises daily and works very hard to a healthy, low cholesterol diet. He has maintained these healthy habits for the past year. He has a father with hypertension. He takes no medications and has no allergies. He does express his desire to avoid medications unless absolutely necessary. Vital signs are normal, blood pressure is 126/82, BMI is 24. Physical exam is unremarkable.

   Labs:

Total cholesterol	256 mg/dL
HDL	50 mg/dL
LDL	170 mg/dL
Triglycerides	180 mg/dL

   Which of the following is the most appropriate management for this patient?
   a. Begin therapy with a low dose statin
   b. Begin therapy with a high dose statin
   c. Obtain lipoprotein(a) level
   d. Repeat lipid screening in 1-2 years

9. According to the USPSTF recommendations, at what age should you begin routine mammogram screening for breast cancer, and how often should you perform routine mammograms for breast cancer screening in adult women?
   a. Begin at age 50, screen yearly
   b. Begin at age 40, screen yearly
   c. Begin at age 50, screen every 2 years
   d. Begin at age 40 and perform screening every 2 years. At age 50, increase frequency to yearly screening.

10. According to the USPSTF recommendations, who should be screened for abdominal aortic aneurysm?
    a. 50-65 year old men that have ever smoked
    b. 65-75 year old men that have ever smoked
    c. 65-75 year old men that have smoked with >10 pack year smoking history
    d. 65-75 year olds (women and men) that have smoked

11. Which of the following statements is true regarding USPSTF recommendations for the frequency of cervical cancer screening with pap smears?
    a. Pap smears should always be performed every 2-3 years if normal, even if HPV testing is negative
    b. If pap smears are normal and HPV testing is negative, women starting at the age of 30 can decrease screening to every 5 years
    c. Pap smears should be performed annually starting at age 21
    d. Even after total abdominal hysterectomy (including cervix removal) for benign indications, pap smears should be performed every 2 years

12. According to the USPSTF recommendations, patients born between 1945 and 1965 specifically should have one-time screening for which of the following?
    a. Syphilis
    b. Hepatitis B
    c. Hepatitis C
    d. HIV

13. A 27-year-old man calls you with a one-day history of right knee pain and swelling, after twisting his knee yesterday playing basketball. Which ONE of the following statements is correct?
    a. The ACL, which connects the femur to the fibula, is commonly injured in athletes.
    b. Patients who note instability of the knee joint usually have nerve impingement in the knee from osteoarthritis.
    c. In the Lachman test, the knee is first fully flexed, followed by forced extension against pressure. Inability to straighten the knee against pressure is a positive test, and represents a torn anterior cruciate ligament.
    d. The McMurray test, in which the flexed knee is passively extended and rotated, is used to evaluate for a torn medial meniscus.

14. In the following list, disorders that present as knee pain are paired with either signs or symptoms. In three of the pairs, signs or symptoms are incorrectly paired with their cause. For which is the sign or symptom correctly paired with the cause?
    a. Patellofemoral syndrome: knee pain worse with leg extended
    b. Bucket-handle medial meniscus tear: patient and examiner unable to fully extend knee
    c. Torn anterior cruciate ligament: leg bends medially when fully extended
    d. Patellar tendonitis: worse with prolonged sitting

15. A 48 year old morbidly obese woman patient presents to your office. She reports significant pain in her left shoulder for the past few weeks. She also reports some weakness in her left arm. On examination, she has anterior shoulder pain when her left arm is extended and she attempts to elevate the arm against resisted force (the speed test). She also has anterior shoulder pain when her left arm is flexed at the elbow to 90 degrees, when attempting to supinate the arm against resistance. During the drop arm test she has mild pain with good control and no weakness. Otherwise her exam is normal, including a normal exam of the right arm and shoulder. She denies other joint symptoms or weakness elsewhere. What is her most likely diagnosis?
    a. Glenohumeral osteoarthritis
    b. Adhesive capsulitis
    c. Biceps tendinopathy/rupture
    d. Rotator cuff tendinopathy or tear

Please circle/answer the questions below.

1. Gender F M

2. Age ___________
3. What is the last month/year of your most recent primary care rotation (Including family medicine, general internal medicine, med/peds clinic)?

   _________/___________
4. How many months of primary care clinic rotations have you had during medical school (including family medicine, general internal medicine, med/peds clinic)?

   ____

I feel confident evaluating and managing…	1 Strongly disagree	2 Disagree	3 Neutral	4 Agree	5 Strongly agree
1. Diabetes in an outpatient clinic setting.	1	2	3	4	5
2. Hypertension in an outpatient clinic setting.	1	2	3	4	5
3. Hyperlipidemia in an outpatient clinic setting.	1	2	3	4	5
4. Recommended routine health maintenance screening in an outpatient clinic setting.	1	2	3	4	5
5. Common musculoskeletal complaints in an outpatient clinic setting.	1	2	3	4	5

	1 Strongly disagree	2 Disagree	3 Neutral	4 Agree	5 Strongly agree
1. I feel that I received sufficient training in primary care during medical school to manage patients independently in a primary care clinic.	1	2	3	4	5
2. I feel that an intern ambulatory boot camp will be good preparation for ambulatory clinic in internal medicine internship.	1	2	3	4	5
3. I feel that I have received sufficient training during medical school in communicating with nurses and clinic staff.	1	2	3	4	5

Thank you for participating!

Internal Medicine Ambulatory Curriculum Immediate Post-Test/Survey

Please answer the following questions to the best of your ability. Each question has one best answer. Please do not write your name on this test. We will be using the anonymous results for research purposes. Participating is completely voluntary. Thanks for your participation!

1. Ms. Smith is an 88 year old woman that you are seeing during a follow up clinic appointment. She has type 2 diabetes mellitus and you have been working with her to get her diabetes optimally controlled. Currently she takes metformin 1000 mg bid. She has been stable on this regimen for 2 years. She also has a history of myocardial infarction and congestive heart failure secondary to coronary artery disease. Her left ventricular ejection fraction is 15%. She is wheel-chair bound and lives at a nursing home. Her hemoglobin A1C checked on today's blood work is 7.8. Her random blood glucose in your office today is 188. Which of the following is the most appropriate statement regarding her hemoglobin A1C goal?
    a. Her goal hemoglobin A1C is <6.5. Another oral medication, such as a sulfonylurea, should be added and the hemoglobin A1C should be rechecked in 3 months.
    b. Her goal hemoglobin A1C is <7.0. Long acting insulin at bedtime should be added and the hemoglobin A1C should be rechecked in 1 month.
    c. Her goal hemoglobin A1C is <7.0. Another oral medication, such as a sulfonylurea, should be added and the hemoglobin A1C should be rechecked in 3 months.
    d. Her goal hemoglobin A1C is <8.0 in the setting of her multiple comorbidities and limited life expectancy. Current management is appropriate. A repeat hemoglobin A1C should be rechecked in 6 months.

2. Which of these comorbid conditions commonly found in diabetes is matched up correctly with the current comorbidity screening recommendations in diabetic (type II) patients?
    a. Nephropathy: Spot urine albumin-to-creatinine ratio every 2 years
    b. Neuropathy: No screening recommended
    c. Retinopathy: Full eye exam every 2 years
    d. None of the above

3. Mr. Kale is a 53 year old man with type 2 diabetes mellitus. He has been on metformin 1000 mg bid and glipizide extended release 20 mg daily for the past 1 year. He tells you at his clinic visit that he is checking his blood glucose every morning before eating. For the past 3 months, his morning glucose values average around 200 and are never below 150. He reports compliance with his medications, as well as a diet and exercise routine. His weight in clinic today is 100 kg. His hemoglobin A1C today is 9.7. Which of the following is the most appropriate next step in management to get his A1C at goal?
    a. Add Januvia (sitagliptin) 100 mg once daily in addition to the metformin and glipizide
    b. Start glargine 20 units at bedtime, continue metformin but discontinue the glipizide
    c. Start glargine 20 units at bedtime, continue the metformin and the glipizide
    d. Start Humalin 70/30 (insulin NPH/insulin regular) 20 units twice daily, continue the metformin and glipizide

4. A 55 year old man comes for an office visit after he was recently diagnosed with type 2 diabetes. He has no history of heart or renal disease. On physical exam, his blood pressure is 138/86 mm Hg, heart rate is 80 bpm, and respiratory rate is 16 breaths/min. His BMI is 29. The remainder of his physical exam is normal. Laboratory studies including electrolytes, creatinine, and BUN are normal. His hemoglobin A1C is 10.0. His urine albumin/creatinine ratio is 20 mg/g. What is the maximal allowable target blood pressure for this patient?
    a. Less than 115/75 mm Hg
    b. Less than 125/75 mm Hg
    c. Less than 130/80 mm Hg
    d. Less than 140/90 mm Hg

5. Ms. Jones comes into your clinic as a new patient. She is a healthy 51 year old woman with a past medical history of osteoarthritis and obesity who was referred to you for elevated blood pressure. She was seen by Gynecology 2 weeks ago for her routine pap smear and was found to have a blood pressure of 162/100, although she felt fine. She was also seen by Rheumatology 3 days ago for evaluation of her osteoarthritis and was found to have an elevated blood pressure at that visit as well, 168/96. Her blood pressure today is 160/92. She reports that she has been checking her blood pressure at home as well and she gets similar readings. You do a very thorough history and physical exam and decide that she likely has primary essential hypertension. Which of the following studies are indicated in the routine evaluation of a patient with newly diagnosed hypertension?
    a. EKG, blood chemistries, lipid panel, urinalysis
    b. Blood chemistries, lipid panel, thyroid studies
    c. Blood chemistries, lipid panel, transthoracic echocardiogram
    d. EKG, blood chemistries, thyroid studies, urinalysis

6. Which type of anti-hypertensive is currently recommended as the first line anti-hypertensive in a nonblack otherwise healthy adult?
    a. Thiazide
    b. Ace inhibitor/Angiotensin receptor blocker
    c. Calcium channel blocker
    d. All of the above

7. A 60 year old man with type 2 DM is seen in PCG to establish medical care. Is other medical problems include HTN and he is a current smoker, smoking 1 pack per day for the past 40 years. His daily medications are metformin, aspirin, and hydrochlorothiazide. On physical examination, his blood pressure is 125/75 mm Hg and his pulse is 80 bpm. His BMI is 27. The remaining physical examination findings are normal. Labs are shown below:

Total cholesterol	160 mg/dL
HDL	60 mg/dL
LDL	64 mg/dL
Triglycerides	180 mg/dL
Hemoglobin A1C	6.5%

Which of the following drugs should be initiated, if any?
    a. No therapy indicated, patient’s LDL is at goal
    b. Simvastain 20 mg daily
    c. Atorvastatin 80 mg daily
    d. Niacin 250 mg daily

8. A 40 year old African American man is evaluated at a follow up visit in your clinic. He had a fasting lipid panel performed 3 weeks ago at your last visit. He does not smoke, has no history of heart attack or stroke, no diabetes. He is a healthy, active man and exercises daily and works very hard to a healthy, low cholesterol diet. He has maintained these healthy habits for the past year. He has a father with hypertension. He takes no medications and has no allergies. He does express his desire to avoid medications unless absolutely necessary. Vital signs are normal, blood pressure is 126/82, BMI is 24. Physical exam is unremarkable.

   Labs:

Total cholesterol	256 mg/dL
HDL	50 mg/dL
LDL	170 mg/dL
Triglycerides	180 mg/dL

   Which of the following is the most appropriate management for this patient?
   a. Begin therapy with a low dose statin
   b. Begin therapy with a high dose statin
   c. Obtain lipoprotein(a) level
   d. Repeat lipid screening in 1-2 years

9. According to the USPSTF recommendations, at what age should you begin routine mammogram screening for breast cancer, and how often should you perform routine mammograms for breast cancer screening in adult women?
   a. Begin at age 50, screen yearly
   b. Begin at age 40, screen yearly
   c. Begin at age 50, screen every 2 years
   d. Begin at age 40 and perform screening every 2 years. At age 50, increase frequency to yearly screening.

10. According to the USPSTF recommendations, who should be screened for abdominal aortic aneurysm?
    a. 50-65 year old men that have ever smoked
    b. 65-75 year old men that have ever smoked
    c. 65-75 year old men that have smoked with >10 pack year smoking history
    d. 65-75 year olds (women and men) that have smoked

11. Which of the following statements is true regarding USPSTF recommendations for the frequency of cervical cancer screening with pap smears?
    a. Pap smears should always be performed every 2-3 years if normal, even if HPV testing is negative
    b. If pap smears are normal and HPV testing is negative, women starting at the age of 30 can decrease screening to every 5 years
    c. Pap smears should be performed annually starting at age 21
    d. Even after total abdominal hysterectomy (including cervix removal) for benign indications, pap smears should be performed every 2 years

12. According to the USPSTF recommendations, patients born between 1945 and 1965 specifically should have one-time screening for which of the following?
    a. Syphilis
    b. Hepatitis B
    c. Hepatitis C
    d. HIV

13. A 27-year-old man calls you with a one-day history of right knee pain and swelling, after twisting his knee yesterday playing basketball. Which ONE of the following statements is correct?
    a. The ACL, which connects the femur to the fibula, is commonly injured in athletes.
    b. Patients who note instability of the knee joint usually have nerve impingement in the knee from osteoarthritis.
    c. In the Lachman test, the knee is first fully flexed, followed by forced extension against pressure. Inability to straighten the knee against pressure is a positive test, and represents a torn anterior cruciate ligament.
    d. The McMurray test, in which the flexed knee is passively extended and rotated, is used to evaluate for a torn medial meniscus.

14. In the following list, disorders that present as knee pain are paired with either signs or symptoms. In three of the pairs, signs or symptoms are incorrectly paired with their cause. For which is the sign or symptom correctly paired with the cause?
    a. Patellofemoral syndrome: knee pain worse with leg extended
    b. Bucket-handle medial meniscus tear: patient and examiner unable to fully extend knee
    c. Torn anterior cruciate ligament: leg bends medially when fully extended
    d. Patellar tendonitis: worse with prolonged sitting

15. A 48 year old morbidly obese woman patient presents to your office. She reports significant pain in her left shoulder for the past few weeks. She also reports some weakness in her left arm. On examination, she has anterior shoulder pain when her left arm is extended and she attempts to elevate the arm against resisted force (the speed test). She also has anterior shoulder pain when her left arm is flexed at the elbow to 90 degrees, when attempting to supinate the arm against resistance. During the drop arm test she has mild pain with good control and no weakness. Otherwise her exam is normal, including a normal exam of the right arm and shoulder. She denies other joint symptoms or weakness elsewhere. What is her most likely diagnosis?
    a. Glenohumeral osteoarthritis
    b. Adhesive capsulitis
    c. Biceps tendinopathy/rupture
    d. Rotator cuff tendinopathy or tear

Please rate the following statements regarding your experience with the intern ambulatory bootcamp from 1-5.

	1<br>Strongly disagree	2<br>Disagree	3<br>Neutral	4<br>Agree	5<br>Strongly agree
6. Intern ambulatory boot camp was good preparation for ambulatory clinic in internal medicine internship.	1	2	3	4	5
7. Educational lectures were engaging.	1	2	3	4	5
8. I learned useful information from the training sessions.	1	2	3	4	5
9. Ambulatory intern boot camp should be a required component of internship.	1	2	3	4	5
10. Lecture sessions on selected ambulatory topics boosted my confidence in managing common conditions encountered in the ambulatory clinic.	1	2	3	4	5
11. The educational sessions will allow me to provide better care for my clinic patients.	1	2	3	4	5
12. Intern ambulatory boot camp helped me learn to communicate with nurses and clinic staff.	1	2	3	4	5

Thank you for participating!

Internal Medicine Ambulatory Curriculum Post-Survey
Completed 2 months after boot camp completion.

Please do not write your name on this. We will be using the anonymous results for research purposes. Participating is completely voluntary. Please circle/answer the questions below.

1. Gender F M
2. Age ______

Please rate the following statements regarding your experience from 1-5.

	1 Strongly disagree with the statement	2 Disagree with the statement	3 Neutral about the statement	4 Agree with the statement	5 Strongly agree with the statement
I feel that I received sufficient training during medical school in communicating with nurses and clinic staff.	1	2	3	4	5
I feel that the intern ambulatory boot camp helped me learn to communicate with nurses and clinic staff.	1	2	3	4	5
The training session on how to effectively use the EMR was good preparation for ambulatory clinic.	1	2	3	4	5
Intern ambulatory boot camp was good preparation for ambulatory clinic in internal medicine internship.	1	2	3	4	5
I used something I learned in ambulatory boot camp in clinic already.	1	2	3	4	5

What was the best part of intern ambulatory boot camp?
____
____
____

What could have been improved about the intern ambulatory boot camp?
____
____
____

What should be added to the intern ambulatory boot camp?
____
____
____

Thank you for participating!
